# Perceived Social Support and Depression, Anxiety, and Stress Symptoms Among Adults Receiving Mental Health Care in Jeddah, Saudi Arabia: A Cross-Sectional Study

**DOI:** 10.3390/healthcare14183069

**Published:** 2026-09-17

**Authors:** Alaa Mahsoon, Faten Jawad, Bashayer Alsulami, Zaina Naser, Fatemah Alzharani, Loujain Sharif

**Affiliations:** 1 Department of Psychiatric and Mental Health Nursing, Faculty of Nursing, King Abdulaziz University, Jeddah 21589, Saudi Arabia; lsharif@kau.edu.sa; 2 King Abdulaziz University Hospital, Ministry of Education, Jeddah 21589, Saudi Arabia; fajawad@kau.edu.sa; 3 Al Nahdi Care Clinic, Jeddah 23545, Saudi Arabia; balsulami91@gmail.com; 4 Faculty of Nursing, King Abdulaziz University, Jeddah 21589, Saudi Arabia; zhamweda@kau.edu.sa; 5 East Jeddah Hospital, Ministry of Health, Jeddah 22334, Saudi Arabia; fatimaner8@gmail.com

**Keywords:** social support, depression, anxiety, stress, psychological, mental health services, Saudi Arabia

## Abstract

**Highlights:**

**What are the main findings?**

- Higher perceived social support was associated with lower depression, anxiety, stress, and total psychological symptoms.
- Family and significant-other support showed higher mean scores than friend support; however, the study did not directly assess stigma, disclosure climate, or emotional safety.

**What are the implications of the main findings?**

- Perceived social support may be a useful psychosocial factor to consider during mental health assessment and care planning.
- Future longitudinal and diagnosis-specific studies are needed to clarify how different sources of support relate to symptom burden over time.

**Abstract:**

Background/Objectives: Perceived social support is associated with psychological well-being, but clinical evidence from adults receiving mental health care in Saudi Arabia remains limited. This study examined associations between perceived social support and self-reported symptoms of depression, anxiety, and stress among adults receiving mental health care in Jeddah, Saudi Arabia. Methods: A cross-sectional analytical study was conducted among 307 adults receiving mental health care in selected Ministry of Health facilities. Participants completed an Arabic self-report questionnaire including sociodemographic variables, the Multidimensional Scale of Perceived Social Support, and the Depression Anxiety Stress Scale-21. Descriptive statistics, internal consistency testing, Spearman correlations, group comparisons, and multivariable linear regression with HC3 robust standard errors were used. Results: The total DASS-21 item-average score was 0.755 ± 0.660, with stress showing the highest mean score. The total MSPSS score was 4.935 ± 1.536. Total perceived social support was inversely associated with total psychological symptoms (rho = −0.229, *p* < 0.001), depression (rho = −0.270, *p* < 0.001), anxiety (rho = −0.223, *p* < 0.001), and stress (rho = −0.157, *p* = 0.006). In the adjusted regression model, total perceived social support remained independently associated with lower total psychological symptom scores (B = −0.111, robust SE = 0.030, 95% CI: −0.170 to −0.052, *p* < 0.001). The model explained 8.4% of the variance in total psychological symptoms. Conclusions: Higher perceived social support was associated with lower self-reported depression, anxiety, and stress symptoms, although the magnitude of associations was small to modest. Findings support considering perceived social support as part of psychosocial assessment in mental health care. These findings should be interpreted cautiously because of the cross-sectional design, convenience sampling, limited clinical characterization, and modest explained variance.

## 1. Introduction

Perceived social support is increasingly recognized as an important psychosocial factor related to mental health outcomes [1,2]. For adults receiving mental health care, perceived social support reflects the individual’s subjective appraisal that emotional, informational, and practical support is available when needed, rather than the mere presence of relatives or friends [3,4]. This distinction is clinically important because patients may have social networks but may not experience these relationships as reliable, supportive, or comfortable for discussing psychological distress.

The relationship between perceived social support and symptoms of depression, anxiety, and stress has been supported by several theoretical perspectives. The stress-buffering model proposes that social support may reduce the psychological burden of stressful experiences by strengthening coping resources and reducing perceived threat [5]. Relational regulation theory further suggests that everyday interactions with close others may help regulate affect and support emotional stability [6]. Previous studies have also suggested possible pathways linking perceived support with psychological symptoms, including perceived stress, sense of coherence, stigma, self-efficacy, self-compassion, and cultural context [7,8,9,10]. However, the present study did not directly test stress-buffering, mediation, perceived stress, stigma, self-efficacy, self-compassion, or treatment-adherence mechanisms; these frameworks were used only to guide interpretation of observed associations.

Depression, anxiety, and stress symptoms are clinically relevant experiences among people receiving mental health care. These symptoms may affect functioning, interpersonal relationships, treatment engagement, and continuity of care, even when they are not the primary diagnosis. Because depression and anxiety symptoms may overlap and interact with relationship-specific support and strain, examining both subscale scores and an overall psychological symptom score may provide useful information about general symptom burden [11]. At the same time, source-specific support may be clinically important because support from family, friends, and significant others may differ in availability, meaning, and perceived usefulness [3,4,11].

The Saudi context adds an important cultural dimension. Mental health help-seeking in Saudi Arabia may be affected by stigma, limited awareness, privacy concerns, and service-related barriers [12]. Therefore, the presence of family support should not be assumed to mean that patients feel comfortable discussing psychological symptoms or seeking emotional help. Although the association between perceived social support and psychological symptoms has been widely studied, evidence from adults receiving mental health care in Saudi Arabia remains limited. This context is important because support may be shaped not only by the availability of relatives or friends, but also by privacy concerns, stigma, family structure, and willingness to disclose psychological distress [12,13]. Therefore, examining family, friend, and significant-other support separately may provide more clinically useful information than treating social support as a single undifferentiated construct [3,13].

This study examined associations between perceived social support and self-reported symptoms of depression, anxiety, and stress among adults receiving mental health care in Jeddah, Saudi Arabia. Specifically, the study addressed the following research questions:

### Research Questions

- What are the levels of perceived social support and self-reported depression, anxiety, and stress symptoms among adults receiving mental health care in Jeddah, Saudi Arabia?
- How are total and source-specific perceived social support associated with depression, anxiety, stress, and total psychological symptom scores?
- Is total perceived social support independently associated with total psychological symptom scores after adjustment for selected sociodemographic covariates?

## 2. Materials and Methods

### 2.1. Design

A cross-sectional analytical design was used. This design was appropriate for estimating levels of perceived social support and psychological symptoms at one point in time and examining the strength and pattern of associations between these variables. The manuscript was prepared in accordance with the Strengthening the Reporting of Observational Studies in Epidemiology (STROBE) guideline for cross-sectional studies [14].

### 2.2. Setting

The study was conducted in selected Ministry of Health mental health facilities in Jeddah, Saudi Arabia. The approved research sites were East Jeddah Hospital, Al-Amal Hospital/Eradah and Mental Health Complex, and King Abdulaziz Hospital Jeddah/Al-Mahjar. Data collection was coordinated through outpatient appointment systems and authorized clinic staff at the participating sites.

### 2.3. Participants and Sampling

Participants were recruited using convenience sampling from selected Ministry of Health mental health facilities; therefore, the sample should not be interpreted as representative of all adults receiving mental health care in Saudi Arabia. Because the study used an electronic self-report questionnaire, participants were required to understand Arabic and be clinically stable enough to complete the survey. Clinical stability was determined by authorized clinic staff based on routine clinical judgment before the questionnaire link was shared. Participants were eligible for inclusion if they were aged 18 years or older, were receiving mental health care from one of the selected facilities, were able to read and understand Arabic, were clinically stable enough to complete a self-report questionnaire, and agreed to participate.

Participants were excluded if they were younger than 18 years, unable to complete the questionnaire because of severe cognitive impairment, intellectual disability, acute confusion, severe medical or neurological limitations, or experiencing an acute psychiatric crisis at the time of data collection, such as severe agitation, severe distress, active psychotic symptoms interfering with questionnaire completion, or immediate suicide risk. A total of 307 participants were included in the final analysis.

The study did not collect psychiatric diagnosis, illness duration, treatment type, medication adherence, previous psychiatric admissions, trauma history, living arrangement, or psychiatric/physical comorbidity.

### 2.4. Sample Size

The minimum required sample size was estimated using an a priori power analysis because the primary objective was to examine the association between perceived social support and self-reported psychological symptoms. The calculation was conducted using G*Power 3.1.9.7 for a two-tailed correlation analysis, with an alpha level of 0.05, statistical power of 0.80, and an expected small-to-moderate effect size of rho = 0.20, consistent with conventional effect-size guidance and G*Power 3.1.9.7 procedures [15,16,17]. The formal a priori power calculation was based on the primary correlation analysis. No separate a priori power calculation was conducted for subgroup comparisons or multivariable regression. However, the final sample of 307 exceeded the minimum required sample for the primary association analysis and allowed exploratory adjusted analyses with a limited number of covariates. Findings from subgroup analyses should therefore be interpreted cautiously.

### 2.5. Study Instruments

Data were collected using a structured self-report questionnaire composed of three sections: sociodemographic characteristics, psychological symptoms, and perceived social support. The Arabic versions of the Depression Anxiety Stress Scale-21 (DASS-21) and the Multidimensional Scale of Perceived Social Support (MSPSS) were used. The MSPSS assesses perceived support from family, friends, and significant others, and Arabic validation studies support its use in Arabic-speaking populations [3,4,18,19]. The DASS-21 has been used to assess self-reported symptoms of depression, anxiety, and stress, and psychometric evidence supports its reliability and validity [20,21,22]. Internal consistency was examined in the current sample using Cronbach’s alpha.

The sociodemographic section collected age group, gender, marital status, employment status, educational level, monthly family income, and parents’ marital status. Psychological symptoms were measured using the DASS-21, a standardized 21-item instrument that measures self-reported symptoms of depression, anxiety, and stress and is not a diagnostic instrument [22]. Participants rated each item on a four-point scale from 0, did not apply to me at all, to 3, applied to me very much or most of the time. Mean scores were calculated for each subscale and the total scale. For the present analyses, DASS-21 depression, anxiety, stress, and total psychological symptom scores were calculated as item-average scores ranging from 0 to 3. This approach was used to place subscale and total scores on a comparable metric. The study did not classify participants into formal DASS-21 severity categories because conventional severity thresholds are usually based on summed subscale scores rather than sample-level item-average scores. Therefore, symptom scores are interpreted descriptively rather than diagnostically. Psychometric evidence supports the reliability and validity of the DASS-21, although the subscales may also reflect a broader psychological distress factor [20,21,22].

Perceived social support was measured using the Multidimensional Scale of Perceived Social Support (MSPSS), a 12-item self-report instrument assessing perceived support from family, friends, and significant others [3]. Each source is represented by four items. Participants responded using a seven-point scale from 1, strongly disagree, to 7, strongly agree. Mean scores were calculated for family, friend, significant other, and total support. In the MSPSS, “significant other” refers to an important person perceived by the respondent as a meaningful source of support. Depending on the respondent’s circumstances, this may include a spouse, partner, close relative, or trusted person. Arabic validation studies support the use of the MSPSS in Arabic-speaking populations [18,19].

### 2.6. Data Collection

Data were collected electronically after ethical approval and site permissions were obtained. Eligible patients were identified through routine appointment lists at the participating mental health facilities. After approval from the relevant department head, clinic nurse, and site research coordinator, patients were contacted by authorised clinic staff before the questionnaire link was shared. The study purpose, voluntary nature of participation, confidentiality procedures, and right to withdraw were explained before questionnaire completion. Participants who agreed to participate received the electronic questionnaire link. The first page of the questionnaire included study information and consent-related information. Completion of the questionnaire was considered confirmation of willingness to participate in the electronic survey. Data collectors were available to clarify questions without influencing responses. Completed responses were reviewed for completeness and stored securely before analysis.

### 2.7. Ethical Considerations

Ethical approval was obtained from the Nursing Research Ethical Committee, Faculty of Nursing, King Abdulaziz University, and from the Local Research Ethics Committee affiliated with the Ministry of Health, Jeddah, Saudi Arabia. The King Abdulaziz University Faculty of Nursing approval was granted by the Chair of the Nursing Research Ethical Committee for the project titled “The impact of family and social support on mental health patients and the increase in the severity of symptoms in Saudi Arabia.” The King Abdulaziz University Faculty of Nursing approval letter did not include a separate approval/reference number. The Ministry of Health Local Research Ethics Committee approval was granted under LEC reference number KSA: H-02-J-002, approval number A02454, dated 19 February 2026, for the project titled “The association between perceived social support and psychiatric symptoms among mental health patients in Jeddah, Saudi Arabia.” Both institutional approvals referred to the same study protocol and the same dataset reported in this manuscript, although the approved study titles differed slightly across committees.

### 2.8. Statistical Analysis

Descriptive analyses, reliability estimates, correlation analyses, and group comparisons were conducted using IBM SPSS Statistics, version 32.0. The de-identified dataset and corresponding variable codebook are provided in Supplementary File S1. Multivariable ordinary least-squares regression models with heteroscedasticity-consistent HC3 robust standard errors and variance inflation factors were computed using Python version 3.12. with the statsmodels package. Data were checked for completeness, coding accuracy, missing values, and distributional characteristics before analysis. Descriptive statistics were used to summarize sociodemographic characteristics, perceived social support, and psychological symptom scores. Internal consistency reliability was assessed using Cronbach’s alpha.

Because the main scale scores were not normally distributed and some subgroup sizes were unequal, Spearman rank-order correlations were used to examine associations between perceived social support and psychological symptoms. Group comparisons were treated as secondary/exploratory analyses. Gender differences were examined after excluding participants who preferred not to disclose gender. Welch’s *t*-tests were used for gender comparisons because group sizes were unequal. Kruskal–Wallis tests were used for age-group comparisons because age groups were highly unequal and distributional assumptions were uncertain.

A multivariable ordinary least-squares linear regression model with heteroscedasticity-consistent HC3 robust standard errors was used to examine whether total perceived social support was independently associated with total psychological symptom scores after adjustment for gender, age group, and monthly family income. Robust standard errors were used to reduce sensitivity to potential heteroscedasticity. Gender, age group, and monthly family income were selected as sociodemographic covariates because of their theoretical and empirical relevance to psychological symptoms and perceived social support. Age group and monthly family income were entered as ordinal variables because they were collected as ordered categories; however, this coding was interpreted cautiously because the age groups were unevenly distributed and income intervals were not equal. Participants with non-disclosed gender and/or unknown or incomplete income coding were excluded from regression analyses using complete-case analysis, resulting in a regression sample of 291 participants. Multicollinearity was assessed using variance inflation factors. To assess the robustness of the main adjusted association to covariate coding, a sensitivity analysis was conducted in which age group and monthly family income were treated as categorical variables using dummy coding, with the youngest age group and lowest income category as reference categories. Influential observations were assessed using Cook’s distance. The distribution of cases excluded from the complete-case regression analysis was also examined.

Secondary adjusted analyses were conducted to examine whether the association between perceived support and psychological symptoms was consistent across DASS-21 subscale outcomes and MSPSS support sources. Separate OLS regression models with HC3 robust standard errors were used for depression, anxiety, and stress outcomes, and for family, friend, and significant-other support variables. These models adjusted for female gender, age group, and monthly family income and were presented as secondary analyses in the Supplementary Materials.

Statistical significance was set at *p* < 0.05. The primary adjusted analysis examined the association between total perceived social support and total psychological symptom scores. Correlation analyses, subgroup comparisons, and supplementary adjusted models were considered secondary/exploratory analyses; therefore, these findings were interpreted cautiously with attention to effect magnitude, consistency of results, and the possibility of Type I error from multiple testing.

## 3. Results

### 3.1. Participant Characteristics

A total of 307 adults receiving mental health care were included in the analysis. The sample was predominantly young, with 133 participants (43.3%) aged 18–24 years and 119 participants (38.8%) aged 25–34 years. Most participants were male (*n* = 242, 78.8%), single (*n* = 196, 63.8%), and employed (*n* = 172, 56.0%). Almost half had university-level education (*n* = 148, 48.2%). Monthly family income was most commonly 3000–5999 Saudi riyals (*n* = 137, 44.6%). Most participants reported that their parents were together and had never separated (*n* = 236, 76.9%) (Table 1).

### 3.2. Psychological Symptoms and Perceived Social Support

The total DASS-21 item-average score was 0.755 (SD = 0.660). Stress had the highest mean score (M = 0.850, SD = 0.692), followed by depression (M = 0.730, SD = 0.712) and anxiety (M = 0.684, SD = 0.701). The mean total perceived social support score was 4.935 (SD = 1.536) on a 1–7 scale. Family support had the highest mean score (M = 5.123, SD = 1.596), followed by significant-other support (M = 5.087, SD = 1.912), while friend support had a lower mean score (M = 4.594, SD = 1.678) (Table 2).

### 3.3. Relationship Between Perceived Social Support and Psychological Symptoms

Spearman correlation analysis showed a statistically significant inverse relationship between perceived social support and psychological symptoms. Higher total perceived social support scores were associated with lower total psychological symptom scores (rho = −0.229, *p* < 0.001). Total perceived social support was also significantly associated with lower depression (rho = −0.270, *p* < 0.001), anxiety (rho = −0.223, *p* < 0.001), and stress (rho = −0.157, *p* = 0.006). Family support showed the numerically largest correlation with total psychological symptoms (rho = −0.226, *p* < 0.001), followed by friend support (rho = −0.190, *p* < 0.001) and significant-other support (rho = −0.188, *p* < 0.001). However, differences between correlation coefficients were not formally tested; therefore, these source-specific correlations should be interpreted descriptively (Table 3).

### 3.4. Sociodemographic Differences

Female participants reported significantly higher anxiety, stress, and total psychological symptom scores than male participants, while the difference in depression scores approached but did not reach statistical significance. The total psychological symptom score was higher among female participants (M = 0.952, SD = 0.592) than male participants (M = 0.714, SD = 0.673; Welch *p* = 0.011). No statistically significant gender differences were observed in total perceived social support or support subscale scores (Table 4).

Descriptive statistics for total psychological symptoms and total perceived social support across age groups are presented in Table 5. Age-group comparisons did not show statistically significant differences in total psychological symptoms or total perceived social support, as confirmed by the Kruskal–Wallis analyses (Table 6).

### 3.5. Multivariable Association Between Perceived Social Support and Psychological Symptoms

In the multivariable ordinary least-squares linear regression model with heteroscedasticity-consistent HC3 robust standard errors, total perceived social support was independently associated with total psychological symptom scores after adjustment for gender, age group, and monthly family income. Higher total perceived social support was associated with lower total psychological symptom scores (B = −0.111, robust SE = 0.030, 95% CI: −0.170 to −0.052, *p* < 0.001). Female gender was also associated with higher total psychological symptom scores (B = 0.212, robust SE = 0.090, 95% CI: 0.035 to 0.388, *p* = 0.019). Age group and monthly family income were not statistically significant covariates. The model was statistically significant overall (*p* < 0.001) and explained 8.4% of the variance in total psychological symptom scores (R^2^ = 0.084; adjusted R^2^ = 0.071) (Table 7).

Variance inflation factors ranged from 1.01 to 1.05, with a maximum VIF of 1.05, indicating no evidence of problematic multicollinearity. Secondary adjusted analyses examining DASS-21 subscale outcomes and source-specific MSPSS subscales are presented in Supplementary Tables S1 and S2. Although statistically significant, the primary adjusted model explained a modest proportion of variance in total psychological symptom scores. Therefore, perceived social support should be interpreted as one psychosocial factor associated with symptom burden rather than as a strong explanatory factor.

The 16 cases excluded from the regression analysis consisted of 12 participants who preferred not to disclose gender and 4 participants with unknown or incomplete monthly family income coding; these categories did not overlap. In the sensitivity model treating age group and monthly family income as categorical dummy-coded variables, the association between total perceived social support and total psychological symptom score remained statistically significant and similar in magnitude to the primary model (B = −0.101, robust SE = 0.032, 95% CI: −0.163 to −0.039, *p* = 0.001; standardized beta = −0.224; R^2^ = 0.105; adjusted R^2^ = 0.073). No case exceeded Cook’s distance of 1.0, suggesting that no single observation had a highly influential effect on the regression model.

## 4. Discussion

This study examined associations between perceived social support and self-reported depression, anxiety, and stress symptoms among adults receiving mental health care in Jeddah, Saudi Arabia. The most notable finding was that higher perceived social support remained independently associated with lower total psychological symptom burden after adjustment for gender, age group, and monthly family income. This association was also consistent across secondary adjusted analyses examining source-specific MSPSS subscales and DASS-21 subscale outcomes, as shown in Supplementary Tables S1 and S2. However, the magnitude of the correlations was small to modest, and the adjusted model explained a modest proportion of variance. Therefore, perceived social support should be interpreted as one relevant psychosocial factor associated with symptom burden rather than as a strong standalone explanation for psychological symptoms. This interpretation is consistent with literature showing that social connectedness and relationship-specific support are related to mental health outcomes, while remaining cautious about causal or temporal pathways in cross-sectional data [1,2,11,13].

The inverse association between perceived support and psychological symptoms is consistent with the stress-buffering model, which proposes that social support may reduce the psychological burden of stressful experiences by strengthening coping resources and reducing perceived threat [5]. Relational regulation theory similarly suggests that everyday interactions with close others may help regulate affect and support emotional stability [6]. However, the present study did not directly test stress-buffering, mediation, perceived stress, stigma, self-efficacy, self-compassion, treatment engagement, medication adherence, or relapse-related mechanisms. Previous studies have examined some of these possible pathways, including perceived stress, sense of coherence, stigma, self-efficacy, self-compassion, and cultural context [7,8,9,10]. Therefore, these mechanisms should be understood as possible explanatory perspectives rather than mechanisms demonstrated by the present study.

The use of both DASS-21 subscale scores and a total psychological symptom score was supported by the overlap among depression, anxiety, and stress symptoms. In this study, stress had the highest item-average score, followed by depression and anxiety. This pattern should be interpreted descriptively rather than diagnostically because the DASS-21 measures self-reported symptoms and does not establish clinical diagnoses. Previous psychometric work supports the use of the DASS-21 for assessing depression, anxiety, and stress symptoms, while also recognizing that these symptom domains may reflect broader psychological distress [20,21,22]. Accordingly, the total DASS-21 score in the present study was interpreted as a general psychological symptom burden score rather than as a diagnostic indicator.

The pattern of source-specific support scores suggests that family and significant-other support may be more available or more strongly perceived than friend support in this sample. This is consistent with the structure of the MSPSS, which distinguishes support from family, friends, and significant others [3]. Recent psychometric evidence also supports the multidimensional structure of the MSPSS, reinforcing the value of examining support sources separately [4]. Evidence from psychiatric outpatient settings similarly shows that support from family, friends, and significant others may differ among people with mental illness [13]. However, in the present study, differences among source-specific mean scores should be interpreted cautiously unless formally tested, and the correlations by source should be understood as descriptive patterns rather than proof that one source is statistically stronger than another.

The Saudi context adds an important cultural dimension to the interpretation of these findings. Family relationships, privacy concerns, religious beliefs, community expectations, and stigma may shape how individuals seek help, disclose distress, and involve others in care [12,23]. Therefore, the presence of family support should not be assumed to mean that patients feel comfortable discussing psychological symptoms or seeking emotional help. However, the MSPSS does not directly measure emotional safety, stigma, fear of judgment, or disclosure climate. Interpretations related to family disclosure and emotional openness should therefore be considered hypotheses for future research rather than direct findings of this study.

The findings also support the relevance of examining perceived support in relation to symptom patterns. Network-based evidence suggests that depression, anxiety, perceived stress, and relationship-specific support and strain may be interconnected [11]. This supports the clinical value of considering both overall psychological symptom burden and source-specific support. Nevertheless, the associations in the present study were small to modest. Therefore, perceived social support should be understood as one psychosocial factor associated with symptom burden, not as a sufficient explanation for depression, anxiety, or stress among adults receiving mental health care.

Female participants reported higher anxiety, stress, and total psychological symptom scores than male participants, while the difference in depression did not reach statistical significance. This finding should be interpreted cautiously because the sample was strongly male-dominated, with only 53 female participants. Therefore, the gender comparison may be unstable and should be considered exploratory. Future studies with more balanced samples are needed to examine gender differences more reliably.

Age-group comparisons did not show statistically significant differences in total psychological symptoms or perceived social support. However, this finding should not be interpreted as evidence that age is unimportant because the age groups were highly unequal and the oldest group included only three participants. Future studies should collect continuous age and recruit sufficient numbers across age groups to examine age-related differences more reliably.

The findings suggest that assessment of perceived support may be useful in psychosocial care planning for adults receiving mental health care. Mental health nurses may consider asking not only whether support is available, but also which sources of support patients perceive as reliable, useful, and comfortable to involve in care planning. These implications should remain focused on psychosocial assessment and individualized care planning because the present study did not directly evaluate family psychoeducation, stigma-reduction interventions, treatment-adherence support, relapse-prevention planning, or peer-support referral as intervention outcomes [12,13,23].

### Limitations

Several limitations should be considered. First, the cross-sectional design prevents causal or temporal interpretation. The study cannot determine whether perceived social support influences psychological symptoms, whether symptom burden affects perceived support, or whether both are shaped by other clinical or social factors. Second, convenience sampling from selected Ministry of Health facilities may have introduced selection bias and limits generalizability to all adults receiving mental health care in Saudi Arabia. In addition, because recruitment was coordinated through clinic appointment systems and authorized clinic staff, perceived pressure to participate or social desirability bias cannot be fully excluded despite the voluntary and anonymous nature of participation. Third, the sample was predominantly young and male, which limits the stability of gender and age-group comparisons. Fourth, patients experiencing acute psychiatric crisis, severe distress, active psychotic symptoms interfering with questionnaire completion, or immediate suicide risk were excluded; this may have resulted in a clinically more stable sample with lower symptom scores. Fifth, the study did not collect psychiatric diagnosis, illness duration, treatment type, pharmacotherapy, medication adherence, previous hospitalization, trauma history, living arrangement, family structure, or comorbid psychiatric or physical illness. These unmeasured factors may confound the association between perceived social support and psychological symptoms. Sixth, DASS-21 scores were self-reported symptom scores and should not be interpreted as clinical diagnoses. Seventh, internal consistency reliability was examined, but structural validity of the DASS-21 and MSPSS was not tested in this clinical sample. Finally, multiple correlations and subgroup analyses were conducted; therefore, secondary findings should be interpreted cautiously.

## 5. Conclusions

Higher perceived social support was associated with lower self-reported depression, anxiety, stress, and total psychological symptom scores among adults receiving mental health care in Jeddah, Saudi Arabia. The associations were statistically significant but small to modest, and the adjusted model explained a limited proportion of variance. Family and significant-other support showed numerically higher mean scores than friend support, but source-specific differences and mechanisms such as stigma, emotional safety, disclosure climate, and treatment adherence were not directly tested. These findings suggest that perceived social support may be a relevant psychosocial domain to consider during clinical assessment and individualized care planning, while highlighting the need for longitudinal, diagnosis-specific, and clinically characterized studies to clarify the role of different support sources in mental health outcomes.

## Figures and Tables

**Table 1:** Sociodemographic Characteristics of Participants (*n* = 307).

Variable	Category	*n*	%
Age group	18–24	133	43.3
	25–34	119	38.8
	35–44	40	13.0
	45–54	12	3.9
	≥55	3	1.0
Gender	Male	242	78.8
	Female	53	17.3
	Prefer not to say	12	3.9
Marital status	Single	196	63.8
	Married	99	32.2
	Divorced	8	2.6
	Widowed	4	1.3
Employment status	Employee	172	56.0
	Student	76	24.8
	Unemployed	46	15.0
	Self-employed	9	2.9
	Retired	4	1.3
Education	Academic (University)	148	48.2
	Secondary	124	40.4
	No formal education	13	4.2
	Advanced study	10	3.3
	Primary	8	2.6
	Middle	4	1.3
Parents’ marital status	Together (never separated)	236	76.9
	One/both deceased	52	16.9
	Separated	19	6.2
Monthly income (SAR)	3000–5999	137	44.6
	<3000	69	22.5
	10,000–14,999	46	15.0
	15,000–24,999	31	10.1
	≥25,000	20	6.5
	Unknown/not stated	4	1.3

Note. Percentages may not total 100 due to rounding. SAR = Saudi Arabian Riyal. No formal education refers to educational attainment and does not indicate inability to read or understand Arabic; all included participants were able to complete the electronic questionnaire independently.

**Table 2:** Descriptive Statistics and Internal Consistency of Scale Scores.

Scale	*n*	Mean	SD	Median	IQR	Cronbach Alpha
Depression (0–3)	307	0.730	0.712	0.571	0.929	0.894
Anxiety (0–3)	307	0.684	0.701	0.429	1.000	0.901
Stress (0–3)	307	0.850	0.692	0.857	0.929	0.876
Total Depression Anxiety Stress Scale-21 (0–3)	307	0.755	0.660	0.667	0.881	0.956
Significant other (1–7)	307	5.087	1.912	5.750	3.250	0.930
Family (1–7)	307	5.123	1.596	5.500	2.500	0.816
Friends (1–7)	307	4.594	1.678	4.750	2.625	0.837
Total Multidimensional Scale of Perceived Social Support (1–7)	307	4.935	1.536	5.250	2.208	0.930

Note. DASS-21 values are item-average scores ranging from 0 to 3. MSPSS values are mean scores ranging from 1 to 7. Higher DASS-21 scores indicate higher self-reported psychological symptoms; higher MSPSS scores indicate higher perceived social support. IQR = interquartile range; SD = standard deviation.

**Table 3:** Spearman Correlations between Perceived Social Support and Psychological Symptoms (*n* = 307).

Support Variable	Symptom Variable	Spearman Rho	*p*
Significant other	Depression	−0.222	<0.001
	Anxiety	−0.184	0.001
	Stress	−0.120	0.036
	Total symptoms	−0.188	<0.001
Family	Depression	−0.263	<0.001
	Anxiety	−0.231	<0.001
	Stress	−0.160	0.005
	Total symptoms	−0.226	<0.001
Friends	Depression	−0.229	<0.001
	Anxiety	−0.182	0.001
	Stress	−0.127	0.027
	Total symptoms	−0.190	<0.001
Total support	Depression	−0.270	<0.001
	Anxiety	−0.223	<0.001
	Stress	−0.157	0.006
	Total symptoms	−0.229	<0.001

Note. Negative values indicate that higher perceived social support was associated with lower psychological symptom scores. These analyses were considered secondary/exploratory and should be interpreted with attention to effect magnitude.

**Table 4:** Gender Differences in Psychological Symptoms and Perceived Social Support.

Variable	Male M (SD), *n* = 242	Female M (SD), *n* = 53	Welch *p*	Cohen d
Depression	0.695 (0.723)	0.903 (0.686)	0.051	0.291
Anxiety	0.635 (0.702)	0.892 (0.644)	0.011	0.372
Stress	0.812 (0.715)	1.062 (0.584)	0.008	0.360
Total symptoms	0.714 (0.673)	0.952 (0.592)	0.011	0.362
Significant other	5.103 (1.868)	5.269 (1.978)	0.579	0.088
Family	5.238 (1.507)	4.892 (1.722)	0.180	−0.224
Friends	4.666 (1.672)	4.575 (1.560)	0.706	−0.055
Total support	5.002 (1.503)	4.912 (1.480)	0.689	−0.060

Note. Gender analysis excluded participants who preferred not to disclose gender. Welch’s test was used for gender comparisons. Cohen’s d is reported as female minus male; positive values indicate higher female scores.

**Table 5:** Age-Group Descriptive Statistics for Total Psychological Symptoms and Total Perceived Social Support.

Age Group	*n*	Total Symptoms M (SD)	Total Support M (SD)
18–24	133	0.766 (0.662)	4.888 (1.551)
25–34	119	0.707 (0.685)	5.108 (1.468)
35–44	40	0.905 (0.621)	4.612 (1.516)
45–54	12	0.615 (0.524)	5.014 (1.754)
≥55	3	0.698 (0.568)	4.083 (2.876)

Note. Total symptom values are DASS-21 item-average scores from 0 to 3. Total support values are MSPSS mean scores from 1 to 7.

**Table 6:** Kruskal–Wallis tests for age-group differences in total psychological symptoms and total perceived social support.

Variable	Kruskal–Wallis H	*p*
Total symptoms	5.195	0.268
Total support	4.043	0.400

Note. Kruskal–Wallis tests were used because age groups were highly unequal, including only three participants aged ≥55 years.

**Table 7:** Multivariable linear regression with robust standard errors for total psychological symptom score.

Variable	B	Robust SE	95% CI	*p*	Standardized Beta
Constant	1.350	0.183	0.991 to 1.709	<0.001	--
Total perceived support	−0.111	0.030	−0.170 to −0.052	<0.001	−0.247
Female gender	0.212	0.090	0.035 to 0.388	0.019	0.123
Age group (ordinal)	−0.004	0.040	−0.083 to 0.075	0.918	−0.006
Monthly family income (ordinal)	−0.027	0.035	−0.094 to 0.041	0.439	−0.046

Note. Model *n* = 291 after excluding participants with non-disclosed gender and/or unknown or incomplete monthly family income coding. The dependent variable was the total DASS-21 item-average psychological symptom score. Female gender was coded as female versus male. Age group and monthly family income were entered as ordinal variables. Robust SEs and 95% CIs were calculated using heteroscedasticity-consistent HC3 standard errors. R^2^ = 0.084; adjusted R^2^ = 0.071; model *p* < 0.001. CI = confidence interval; DASS-21 = Depression Anxiety Stress Scale-21; SE = standard error.

## Data Availability

A de-identified minimal dataset and codebook are provided as Supporting Information files (S1 Dataset and S1 Codebook). These files contain the values needed to reproduce the reported descriptive statistics, reliability estimates, correlation analyses, group comparisons, and regression models. No directly identifying participant information is included.

## Supplementary Materials

The following supporting information can be downloaded at: https://www.mdpi.com/article/10.3390/healthcare14183069/s1, Supplementary File S1, Dataset and Codebook; Supplementary File S2, Secondary adjusted regression analyses, including Table S1, Source-specific adjusted associations between perceived social support and total psychological symptom score, and Table S2, Adjusted associations between total perceived social support and DASS-21 subscale scores.

## Abbreviations

The following abbreviations are used in this manuscript:

CI	confidence interval
DASS-21	Depression Anxiety Stress Scale-21
IQR	interquartile range
MSPSS	Multidimensional Scale of Perceived Social Support
SAR	Saudi Arabian Riyal
SD	standard deviation
SE	standard error
STROBE	Strengthening the Reporting of Observational Studies in Epidemiology
