# Supplementary material for: Perceived Social Support and Depression, Anxiety, and Stress Symptoms Among Adults Receiving Mental Health Care in Jeddah, Saudi Arabia: A Cross-Sectional Study
Source: Healthcare (Basel). 2026 Sep 17;14(18):3069. doi: 10.3390/healthcare14183069 (PMC13606063; doi:10.3390/healthcare14183069)
Supplement: Supplementary file 1 [file healthcare-14-03069-s001.zip › healthcare-4540777-File S2.pdf]

Supplementary Tables S1 and S2

Secondary adjusted regression analyses

Analytic approach: Models were ordinary least-squares linear regression models with HC3 heteroscedasticity-consistent robust standard errors. Complete-case analysis was used (n = 291). Each model adjusted for female gender, age group, and monthly family income. DASS-21 outcomes are item-average scores from 0 to 3; MSPSS variables are mean scores from 1 to 7.

Table S1. Source-specific adjusted associations between perceived social support and total psychological symptom score.

Model	Outcome	Adjusted support variable	B	Robust SE	95% CI	p	Beta	R2	Adjusted R2
S2a	Total psychological symptoms	Family support	-0.097	0.028	-0.152 to -0.042	<0.001	-0.224	0.073	0.060
S2b	Total psychological symptoms	Friend support	-0.087	0.027	-0.139 to -0.035	0.001	-0.214	0.069	0.056
S2c	Total psychological symptoms	Significant-other support	-0.077	0.023	-0.123 to -0.031	0.001	-0.215	0.069	0.056

Note. Each row represents a separate adjusted model. The dependent variable was total DASS-21 item-average psychological symptom score. Covariates were included in the models but are not displayed to focus on source-specific support coefficients. VIF values across S2 models ranged from 1.011 to 1.054, indicating no evidence of problematic multicollinearity. CI = confidence interval; DASS-21 = Depression Anxiety Stress Scale-21; MSPSS = Multidimensional Scale of Perceived Social Support; SE = standard error; VIF = variance inflation factor.

Table S2. Adjusted associations between total perceived social support and DASS-21 subscale scores.

Model	Outcome	Adjusted support variable	B	Robust SE	95% CI	p	Beta	R2	Adjusted R2
S3a	Depression	Total perceived support	-0.140	0.032	-0.203 to -0.078	<0.001	-0.288	0.101	0.089
S3b	Anxiety	Total perceived support	-0.098	0.030	-0.157 to -0.038	0.001	-0.206	0.071	0.059
S3c	Stress	Total perceived support	-0.095	0.032	-0.158 to -0.032	0.003	-0.202	0.059	0.046

Note. Each row represents a separate adjusted model. Total perceived social support was the main independent variable in each model. Covariates were included in the models but are not displayed. VIF values across S3 models ranged from 1.013 to 1.053, indicating no evidence of problematic multicollinearity. CI = confidence interval; DASS-21 = Depression Anxiety Stress Scale-21; MSPSS = Multidimensional Scale of Perceived Social Support; SE = standard error; VIF = variance inflation factor.
